# Literary Notes

**Published:** 1895-09

**Authors:** 


### Literary Notes.

The *Buffalo Druggist* is the name of a new journal that has recently appeared. It is published in Buffalo in the interests of pharmacy and the general drug trade and is well edited. In its issue for July, 1895, it published a fine photograph of Dr. Ernest Wende, one of the associate editors of the Buffalo Medical Journal. Dr. Wende's picture is accompanied by an interesting biographical sketch.

THE *Medical and Surgical Reporter* that has been published in Philadelphia since 1853, when it was established by Dr. S. W. Butler, has removed to New York and installed itself in the new Constable building, 109-111 Fifth avenue, corner Eighteenth. Here, both the editorial and business offices will henceforth be located. We are glad to have such an eminently respectable journal, and one so long and permanently established, take up its residence within the borders of the Empire State.

---

THE *Nashville Journal of Medicine and Surgery*, in its issue for August, appears enlarged in size and with an illuminated cover in commemoration of the founding of the University of Nashville forty-five years ago, and which will move into a handsome new building October 1, 1895.

We beg to tender our most cordial congratulations to our splendid contemporary for the beauty and excellence of its August number.

---

THE *Atlantic Medical Weekly*, published in Providence, R. I., is one of the sprightliest and best of the weekly medical journals that come to our exchange table. It is distinguished for its original articles, but particularly for its interesting editorial department. It is distinctive in its arrangement of medical news under three several heads—namely, state, American and foreign. We predict a brilliant future for this deserving medical journal.

---

THE *Woman's Medical Journal* (Toledo, O.), the only woman's medical journal in the world, in its issue for August, 1895, has begun a series of illustrated biographical sketches of the leading women of the medical profession. The first portrait to greet us is that of Dr. Mary A. Spink, of Indianapolis, Ind. If all the women doctors present as handsome an appearance both of healthful and womanly beauty as Dr. Spink, the gallery of portraits will be well worth preserving.

The JOURNAL takes off its hat in its most courteous manner to the *Woman's Medical Journal.*

---

HALL OF THE COLLEGE OF PHYSICIANS, PHILADELPHIA.—The William F. Jenks memorial prize of $500, under the deed of trust of Mrs. William F. Jenks, has been awarded to A. Brothers, M. D.,

162 Madison street, New York, for the best essay on Infant Mortality During Labor and its Prevention.

The prize committee also reports as highly meritorious the essay on the same subject bearing the motto, *Vade Mecum*.

The writers of the unsuccessful essays can have them returned to any address they may name, by sending it and the motto which distinguished the essay to the chairman of the prize committee; Horace Y. Evans, M. D., College of Physicians, Philadelphia. James V. Ingham, Charles S. Wurts and I. Minis Hays, trustees of the Wm. F. Jenks memorial fund.

---

KEIL'S MEDICAL, PHARMACEUTICAL AND DENTAL DIRECTORY.—George Keil, 1715 Willington street, Philadelphia, announces the early publication (fourth edition) of the Medical and Dental Register Directory and Intelligencer, for the states of Pennsylvania, New York, New Jersey, Maryland, Delaware and the District of Columbia. It will present not only a complete list of all medical and dental practitioners in the states named, with place and date of graduation, but also lists of professional educational institutions, hospitals, asylums and the like, and will be of much practical value to all members of these professions.

---

A. L. KOURSH COMPANY, Chicago, publish a complete case recorder for use in general medicine and gynecology, especially adapted to the necessities of physicians, students, dispensaries and hospitals. It is prepared by S. B. Lyon, M. D., and can be had on application to the publishers and remittance of 20 cents for a complete copy, or $1 for separate blanks in blocks of fifty each.

